# Supplementary material for: An ELISA Using Synthetic Mycolic Acid-Based Antigens with DIVA Potential for Diagnosing Johne’s Disease in Cattle
Source: Animals (Basel). 2024 Mar 9;14(6):848. doi: 10.3390/ani14060848 (PMC10967296; doi:10.3390/ani14060848)
Supplement: Supplementary file 1 [file animals-14-00848-s001.zip › animals-2826460-supplementary.pdf]

Supplementary information

An ELISA using synthetic mycolic acid based antigens with DIVA potential for diagnosing for Johne's disease in cattle

Paul S. Mason, Thomas Holder, Natasha Robinson, Brendan Smith, Rwoa'a T. Hamed, Juma'a R. Al Dulayymi, Valerie Hughes, Karen Stevenson, Gareth J. Jones, H. Martin Vordermeier, Shawn Mc Kenna, and Mark S. Baird

Table S1: ELISA responses to six synthetic lipid antigens and sample information for PEI samples, together with faecal PCR cycle counts and responses in a commercial IDEXX MAP assay (0.55 recommended cut-off for positive).

MAP Faecal PCR Cycles	MAP IDEXX	Serum ID	map class	MOD171	JRRR121	RT237F2	SMP70	ZAM295	ST123
				% Pooled Pos Absorbance					
26.7	2.82	PEI 123	PEI++	14.4	20.6	51.9	112.2	92.6	74.7
22.5	2.77	PEI 48	PEI++	113.5	112.5	66.5	102.0	98.8	82.9
23.1	2.65	PEI 144	PEI++	18.5	71.9	49.9	102.2	95.4	84.5
20.7	2.33	PEI 217	PEI++	33.9	132.5	45.6	76.6	97.6	88.5
25.5	2.32	PEI 103	PEI++	117.3	111.7	78.8	93.3	90.2	66.0
23.9	2.28	PEI 64	PEI++	36.4	52.0	74.6	61.4	97.1	94.7
26.3	2.26	PEI 163	PEI++	24.8	113.9	60.4	108.9	99.6	97.6
24.3	2.18	PEI 114	PEI++	61.7	35.9	64.2	94.3	94.8	47.4
27.3	2.15	PEI 9	PEI++	29.8	26.6	73.0	89.3	109.3	85.0
29.6	1.88	PEI 122	PEI++	34.0	78.3	59.2	88.4	100.1	100.1
24.3	1.72	PEI 46	PEI++	61.1	40.3	39.2	74.3	97.4	75.1
30.1	1.61	PEI 73	PEI++	23.6	37.4	63.0	64.5	97.5	95.8
33.8	1.49	PEI 208	PEI++	17.0	17.2	49.5	42.6	69.3	27.5
34.9	1.15	PEI 200	PEI++	16.8	25.6	53.8	61.1	89.1	29.1
36.2	0.82	PEI 139	PEI++	20.0	32.5	48.2	88.4	77.1	43.1
31.8	0.56	PEI 173	PEI++	13.8	16.7	43.3	74.8	93.8	33.7
35.1	0.19	PEI 4	PEI+-	17.7	18.4	39.2	50.5	90.5	44.3
36.4	0.14	PEI 224	PEI+-	16.5	16.5	61.5	49.3	70.2	16.7
33.2	0.11	PEI 134	PEI+-	12.1	18.8	36.7	64.4	74.6	24.9
33.7	0.07	PEI 69	PEI+-	28.3	66.7	73.8	74.6	61.5	27.8
36.4	0.07	PEI 165	PEI+-	23.5	27.7	51.4	54.4	45.9	21.7
34.7	0.06	PEI 82	PEI+-	17.7	20.0	86.9	61.5	99.5	98.5
35.5	0.05	PEI 2	PEI+-	17.9	28.5	121.7	35.9	69.0	39.5
34.6	0.05	PEI 1	PEI+-	17.2	45.1	56.2	49.8	91.8	72.8
35.9	0.05	PEI 83	PEI+-	18.6	22.6	54.3	64.4	80.8	50.1
36.7	0.05	PEI 18	PEI+-	27.2	22.8	90.0	56.7	66.8	52.0
36.2	0.04	PEI 96	PEI+-	20.5	16.3	56.6	62.4	66.7	39.7

36.4	0.04	PEI 97	PEI+-	43.4	30.6	75.5	75.0	67.6	19.6
35.9	0.03	PEI 35	PEI+-	27.7	21.8	56.4	55.6	44.6	14.2
36.3	0.02	PEI 152	PEI+-	49.5	60.3	81.7	69.1	80.4	32.0
36.1	0.02	PEI 38	PEI+-	21.1	23.2	72.0	77.2	79.7	36.7
36.7	0.02	PEI 70	PEI+-	24.7	22.5	141.9	83.4	91.8	59.0
35.8	0.02	PEI 74	PEI+-	18.2	20.8	46.6	79.1	81.0	20.6
36.4	0.01	PEI 20	PEI+-	22.4	15.5	42.9	55.6	25.2	12.0
36.7	0.01	PEI 54	PEI+-	22.6	30.7	60.9	45.5	66.5	22.7
36.7	0.11	PEI 91	PEI+-	25.9	16.6	160.3	94.0	96.4	83.3
37.1	0.01	PEI 196	PEI+-	22.2	29.8	40.7	86.5	68.7	34.8
37.1	0.03	PEI 183	PEI+-	9.7	17.3	38.7	42.6	33.2	11.0
37.2	0.02	PEI 100	PEI+-	37.3	15.7	48.0	55.7	41.8	24.0
37.2	0.10	PEI 150	PEI+-	22.4	24.7	61.4	82.7	88.8	28.6
37.2	0.04	PEI 157	PEI+-	24.7	29.6	54.0	32.8	64.1	17.8
37.2	0.01	PEI 205	PEI+-	16.6	18.3	83.0	51.1	42.9	14.2
37.3	0.26	PEI 31	PEI+-	22.7	28.1	71.7	53.3	95.1	44.6
37.3	0.06	PEI 8	PEI+-	20.3	18.9	69.3	71.6	89.6	38.7
37.5	0.03	PEI 79	PEI+-	48.5	33.4	80.6	35.6	50.4	19.7
37.7	0.01	PEI 155	PEI+-	10.1	18.7	35.1	73.8	28.4	10.5
37.7	0.05	PEI 15	PEI+-	61.4	40.0	84.5	71.6	68.3	21.7
37.8	0.04	PEI 87	PEI+-	92.9	30.7	103.4	96.0	100.3	95.6
37.9	0.02	PEI 109	PEI+-	54.5	16.4	50.7	108.1	61.0	65.6
37.9	0.12	PEI 228	PEI+-	16.6	13.9	36.1	68.4	32.8	15.5
38.2	0.05	PEI 102	PEI+-	19.1	11.6	34.0	78.0	22.1	12.9
38.2	0.02	PEI 162	PEI+-	12.6	30.2	41.9	55.1	78.8	37.5
38.2	0.09	PEI 111	PEI+-	26.2	13.4	35.1	48.5	48.2	38.1
38.3	0.03	PEI 185	PEI+-	17.0	21.7	40.3	50.9	34.8	15.6
38.3	0.04	PEI 121	PEI+-	21.9	16.2	42.5	65.9	47.6	20.2
38.4	0.01	PEI 168	PEI+-	20.1	18.7	46.5	65.9	35.3	19.2
38.4	0.07	PEI 40	PEI+-	37.7	19.9	64.2	66.2	92.4	43.2
38.5	0.02	PEI 127	PEI+-	35.3	30.0	47.9	63.5	60.8	66.2
38.5	0.01	PEI 174	PEI+-	18.8	14.7	41.6	55.1	37.1	12.9
38.5	0.02	PEI 60	PEI+-	31.3	23.6	43.4	64.4	23.5	18.1
38.5	0.10	PEI 193	PEI+-	77.5	19.6	161.9	86.9	98.9	72.8
38.5	0.03	PEI 133	PEI+-	32.3	12.9	82.1	57.0	39.0	30.9
38.5	0.07	PEI 141	PEI+-	14.8	20.4	43.6	73.6	82.4	28.7
38.5	0.22	PEI 158	PEI+-	23.5	21.2	83.0	69.1	91.9	43.0
38.6	0.09	PEI 167	PEI+-	22.7	20.8	94.0	60.6	53.6	23.5
38.6	0.04	PEI 136	PEI+-	25.4	20.1	58.4	72.3	95.6	94.8
38.6	0.02	PEI 203	PEI+-	17.5	57.9	58.3	25.7	44.3	16.5
38.7	0.03	PEI 10	PEI+-	28.5	18.9	45.6	58.7	34.4	17.0
38.7	0.05	PEI 170	PEI+-	16.9	31.4	56.2	52.9	104.7	89.4
38.9	0.13	PEI 215	PEI+-	33.2	19.1	71.3	97.0	49.5	25.6
38.9	0.04	PEI 44	PEI+-	30.1	45.2	96.3	69.7	58.8	41.6
39.2	0.13	PEI 68	PEI+-	45.9	22.9	56.0	83.8	74.7	52.0
39.2	0.01	PEI 93	PEI+-	41.4	21.2	68.6	43.6	36.2	26.4

39.5	0.16	PEI 221	PEI+-	24.8	23.7	52.6	67.9	93.4	48.7
39.6	0.02	PEI 130	PEI+-	23.1	19.9	33.0	55.0	98.1	86.0
39.6	0.13	PEI 72	PEI+-	40.3	20.6	68.1	28.0	38.6	31.5
39.9	0.04	PEI 26	PEI+-	36.5	34.1	71.0	59.3	58.3	26.7
40.0	0.04	PEI 153	PEI+-	23.9	50.2	54.3	68.1	76.8	44.5
40.2	0.07	PEI 71	PEI+-	56.7	43.5	94.5	53.7	43.5	32.4
41.0	0.01	PEI 6	PEI+-	39.0	15.1	52.8	76.4	47.6	19.3
0	0.03	PEI7	PEI F5	25.0	18.8	48.2	109.8	62.5	23.8
0	0.03	PEI11	PEI F5	42.5	28.7	44.0	94.5	60.2	19.6
0	0.02	PEI12	PEI F5	29.9	53.0	96.0	85.6	64.5	21.1
0	0.06	PEI13	PEI F5	39.4	19.2	54.6	54.1	82.1	55.1
0	0.07	PEI14	PEI F5	20.9	53.7	63.3	49.6	46.7	18.4
0	0.06	PEI30	PEI F5	28.7	21.0	48.8	37.6	55.0	29.6
0	0.05	PEI34	PEI F5	17.2	14.9	42.4	30.1	21.7	12.7
0	0.06	PEI37	PEI F5	32.5	22.0	69.7	54.3	55.0	36.5
0	0.05	PEI39	PEI F5	18.7	15.1	39.4	31.3	50.8	26.4
0	0.17	PEI41	PEI F5	22.4	26.8	35.4	65.3	52.9	22.5
0	0.03	PEI42	PEI F5	27.4	16.2	61.8	46.3	64.2	44.6
0	0.16	PEI52	PEI F5	43.6	21.2	72.4	54.5	96.8	72.0
0	0.13	PEI58	PEI F5	21.7	19.0	52.4	32.0	99.8	32.3
0	0.06	PEI85	PEI F5	19.5	22.4	48.7	41.1	66.2	36.6
0	0.13	PEI94	PEI F5	27.9	18.2	97.5	57.9	49.0	17.0
0	0.03	PEI95	PEI F5	15.6	15.9	43.2	26.5	42.1	19.0
0	0.09	PEI99	PEI F5	15.8	16.3	55.9	35.3	73.3	53.5
0	0.10	PEI101	PEI F5	14.5	15.3	59.6	29.9	74.1	53.5
0	0.11	PEI126	PEI F5	19.0	19.4	52.7	40.3	41.8	13.0
0	0.06	PEI128	PEI F5	32.3	38.1	65.5	49.0	31.6	17.9
0	0.02	PEI129	PEI F5	22.0	24.1	40.3	46.0	40.4	26.0
0	0.03	PEI154	PEI F5	18.5	19.7	45.3	31.9	38.6	16.5
0	0.03	PEI156	PEI F5	26.4	19.3	56.1	42.3	31.8	16.4
0	0.08	PEI160	PEI F5	26.7	29.9	64.0	64.6	72.7	24.3
0	0.08	PEI161	PEI F5	27.7	16.6	66.5	76.5	35.9	20.0
0	0.10	PEI164	PEI F5	22.2	19.4	69.3	37.5	24.6	13.0
0	0.13	PEI181	PEI F5	20.8	18.0	59.4	43.3	28.1	12.5
0	0.08	PEI182	PEI F5	33.7	22.1	71.2	63.3	45.6	20.6
0	0.05	PEI184	PEI F5	31.3	40.1	50.6	63.9	40.0	21.6
0	0.12	PEI186	PEI F5	27.2	19.6	52.2	44.6	43.6	18.7
0	0.18	PEI187	PEI F5	23.8	16.0	48.9	38.6	38.6	16.8
0	0.08	PEI189	PEI F5	31.8	20.5	57.4	35.0	54.7	25.7
0	0.07	PEI211	PEI F5	26.9	18.8	54.0	41.8	63.5	36.7
0	0.08	PEI171	PEI F5	22.2	17.4	51.7	41.3	23.4	14.0
0	0.14	PEI192	PEI F5	24.6	18.9	72.8	42.0	50.7	31.4
0	0.04	PEI197	PEI F5	20.6	20.2	78.4	80.6	40.7	21.1
0	0.07	PEI212	PEI F5	18.1	19.1	45.4	33.0	53.2	18.0
0	0.03	PEI213	PEI F5	25.2	18.5	75.4	43.6	60.3	22.4
0	0.23	PEI216	PEI F5	20.2	17.0	49.7	28.0	74.8	22.1

0	0.02	PEI218	PEI F5	62.1	24.0	63.1	44.9	44.4	19.0
0	0.05	PEI 17	PEI F6	34.0	14.8	37.4	61.6	55.1	25.4
0	0.23	PEI 19	PEI F6	24.8	15.2	35.8	69.1	75.9	34.9
0	0.02	PEI 21	PEI F6	132.1	13.6	39.4	84.3	34.9	14.4
0	0.02	PEI 24	PEI F6	21.3	12.2	35.9	48.5	32.8	16.6
0	0.07	PEI 25	PEI F6	53.5	25.6	141.9	52.5	39.9	14.3
0	0.08	PEI 29	PEI F6	56.4	15.7	44.1	66.6	54.2	29.8
0	0.03	PEI 47	PEI F6	27.8	15.3	45.1	53.2	42.9	13.9
0	0.02	PEI 49	PEI F6	21.3	12.8	36.7	34.6	16.2	11.1
0	0.11	PEI 51	PEI F6	24.4	16.4	54.1	63.7	47.7	20.6
0	0.03	PEI 53	PEI F6	39.9	12.8	49.7	63.2	55.6	36.4
0	0.10	PEI 55	PEI F6	23.9	12.3	39.1	50.3	73.5	24.8
0	0.09	PEI 57	PEI F6	19.5	13.5	29.0	26.0	52.9	34.0
0	0.17	PEI 59	PEI F6	42.7	17.7	57.6	71.8	98.3	82.5
0	0.01	PEI 78	PEI F6	26.3	15.4	45.0	45.8	23.1	13.2
0	0.06	PEI 81	PEI F6	55.9	14.2	48.3	58.1	32.7	21.8
0	0.06	PEI 84	PEI F6	27.3	15.2	59.2	53.9	36.8	23.6
0	0.03	PEI 86	PEI F6	24.2	14.9	46.9	33.0	27.1	16.5
0	0.18	PEI 88	PEI F6	59.0	14.7	45.2	72.9	52.4	30.3
0	0.19	PEI 108	PEI F6	34.2	20.9	60.7	62.2	82.1	67.2
0	0.13	PEI 110	PEI F6	44.1	14.9	54.5	72.2	69.8	66.4
0	0.05	PEI 115	PEI F6	64.7	16.4	42.8	63.5	88.7	70.9
0	0.04	PEI 117	PEI F6	56.1	22.6	47.9	67.4	33.2	22.5
0	0.03	PEI 119	PEI F6	27.7	13.9	36.6	51.9	39.7	35.1
0	0.11	PEI 143	PEI F6	16.2	17.0	42.7	70.7	84.7	44.6
0	0.02	PEI 146	PEI F6	12.0	11.6	31.2	82.1	95.3	63.7
0	0.02	PEI 148	PEI F6	9.3	16.2	36.8	82.3	93.6	44.8
0	0.02	PEI 149	PEI F6	18.1	38.1	75.5	52.2	54.2	29.7
0	0.14	PEI 169	PEI F6	13.6	15.8	46.7	95.3	49.0	26.2
0	0.02	PEI 175	PEI F6	14.2	17.3	46.4	74.0	37.1	19.1
0	0.15	PEI 176	PEI F6	16.4	15.5	46.1	86.4	79.6	77.2
0	0.04	PEI 177	PEI F6	12.6	10.9	37.6	43.7	72.8	53.2
0	0.02	PEI 178	PEI F6	36.5	15.7	50.1	104.8	90.7	69.5
0	0.04	PEI 180	PEI F6	33.4	28.2	51.7	92.9	84.8	60.4
0	0.24	PEI 199	PEI F6	21.0	21.2	46.8	81.5	68.7	53.7
0	0.09	PEI 202	PEI F6	20.7	35.0	36.3	58.9	32.5	21.4
0	0.04	PEI 204	PEI F6	19.7	15.8	82.2	65.4	38.4	24.2
0	0.14	PEI 207	PEI F6	21.3	13.8	42.6	90.1	42.4	19.2
0	0.26	PEI 209	PEI F6	14.4	14.8	36.3	87.4	40.2	20.9
0	0.02	PEI 222	PEI F6	24.8	39.2	34.2	90.0	50.1	30.1
0	0.03	PEI 225	PEI F6	13.6	15.2	38.2	63.3	26.4	13.8

ELISA responses to six synthetic lipid antigens and sample information for PEI samples, together with faecal PCR cycle counts and responses in a commercial IDEXX MAP assay 0.55 (recommended cut-off for positive). A faecal PCR score of 0 corresponds to no response after 50 cycles.

Table S2: ELISA responses (% positive control) to six synthetic lipid antigens and sample information for APHA timeline samples (10 animals at 9 times). Columns 2 and 3 are results for commercial Bovigam IGRA and ParaTB IDEXX assays.

Serum ID	PPDA* Bovigam	ParaTB IDEXX** (S/P, %)	MOD171	JRRR121	RT237F2	SMP70	ZAM295	ST123
			% Pooled Pos Absorbance					
AP2176, Wk0	0.070	6	24.7	24.3	65.0	48.0	89.5	73.6
AP2176, Wk4	0.508		21.0	23.3	75.9	29.2	85.9	46.5
AP2176, Wk8	0.084		25.3	25.1	56.8	61.6	69.6	25.0
AP2176, Wk16	0.405	5	29.0	26.1	50.8	50.9	53.3	11.9
AP2176, Wk24	0.829	3	31.7	24.7	49.3	91.5	97.7	74.4
AP2176, Wk32	2.310		34.2	21.3	27.2	51.2	75.9	10.8
AP2176, Wk40	0.116	4	26.2	22.2	34.9	50.7	48.6	8.7
AP2176, Wk45	1.000	7	26.1	24.3	28.1	41.1	54.0	9.4
AP2176, Wk47		10	28.6	23.6	26.7	41.8	57.1	8.9
AP2169, Wk0	1.063	8	24.5	21.8	35.4	27.8	59.9	9.5
AP2169, Wk4	0.622		21.2	24.7	35.8	22.5	31.9	7.8
AP2169, Wk8	0.791		26.7	26.6	38.4	36.5	33.4	8.8
AP2169, Wk16	1.341	4	27.3	26.4	36.5	58.9	64.7	10.6
AP2169, Wk24	0.839	4	31.2	26.1	40.4	78.9	88.2	24.7
AP2169, Wk32	1.693		30.8	23.2	28.0	87.4	95.6	67.1
AP2169, Wk40	2.863	5	65.7	26.7	29.7	95.8	91.7	46.4
AP2169, Wk45	2.657	9	47.2	29.2	25.7	98.8	94.9	41.4
AP2169, Wk47		8	38.9	22.9	26.3	99.6	92.1	39.6
AP2170, Wk0	0.504	6	22.9	25.0	71.5	45.7	88.8	23.3
AP2170, Wk4	0.054		23.0	25.6	61.3	30.8	70.0	14.8
AP2170, Wk8	0.036		31.2	26.4	57.9	37.0	50.1	12.0
AP2170, Wk16	0.041	5	30.6	24.0	38.6	39.1	59.6	9.4
AP2170, Wk24	0.021	3	28.3	26.2	40.6	34.1	74.9	10.9
AP2170, Wk32	0.349		26.0	29.5	28.5	49.4	68.5	10.7
AP2170, Wk40	-0.133	4	26.1	27.8	25.0	62.4	48.3	9.8
AP2170, Wk45	0.009	7	24.0	22.9	23.2	54.8	47.1	9.5
AP2170, Wk47		10	23.0	27.5	21.9	53.0	53.7	10.0
AP2174, Wk0	0.049	11	22.1	25.9	30.8	30.0	59.3	8.7
AP2174, Wk4	0.075		24.6	26.1	41.8	24.6	33.0	10.0
AP2174, Wk8	0.097		30.5	25.4	44.8	34.3	44.3	9.6
AP2174, Wk16	0.378	9	34.4	27.1	61.0	59.8	39.4	10.3
AP2174, Wk24	1.391	20	44.1	29.3	37.6	41.4	71.4	25.6
AP2174, Wk32	0.057		32.9	26.7	31.7	53.8	80.8	25.6
AP2174, Wk40	0.110	21	28.0	25.1	25.1	43.4	51.2	13.5
AP2174, Wk45	0.857	9	29.0	26.6	24.9	44.8	56.8	11.9
AP2174, Wk47		31	32.3	30.8	27.8	46.1	71.6	15.4
AP2180, Wk0	-0.051	4	22.9	30.1	43.2	34.7	53.4	13.8
AP2180, Wk4	0.058		24.1	23.5	31.3	37.1	30.7	10.9
AP2180, Wk8	0.000		27.3	27.0	33.4	86.1	32.9	10.3

AP2180, Wk16	-0.001	12	36.1	34.9	39.8	70.9	97.5	75.7
AP2180, Wk24	0.030	7	32.4	28.7	34.1	40.4	51.2	10.7
AP2180, Wk32	0.415		26.2	27.8	25.6	70.9	84.9	44.8
AP2180, Wk40	0.019	6	29.5	30.9	24.6	78.3	81.7	52.2
AP2180, Wk45	0.109	8	30.3	33.4	26.1	72.5	72.5	34.5
AP2180, Wk47		13	29.0	34.4	25.9	74.2	73.5	35.8
AP2183, Wk0	-0.021	12	24.1	41.9	222.8	22.7	33.4	10.3
AP2183, Wk4	1.980		22.2	28.8	170.1	26.9	27.7	8.9
AP2183, Wk8	0.109		24.2	28.3	110.4	26.2	39.0	8.8
AP2183, Wk16	0.656	8	26.9	29.3	54.5	32.0	70.7	10.4
AP2183, Wk24	0.409	5	29.8	26.8	35.6	31.8	44.0	10.0
AP2183, Wk32	0.040		22.4	26.3	37.1	28.7	40.6	8.6
AP2183, Wk40	0.018	12	21.6	22.9	28.4	29.4	38.6	8.0
AP2183, Wk45	0.296	7	31.6	27.0	30.5	32.8	43.8	9.7
AP2183, Wk47		5	46.9	26.2	39.3	35.6	47.1	11.0
AP2184, Wk0	0.298	10	19.6	24.4	49.1	47.3	37.0	9.3
AP2184, Wk4	0.426		20.5	22.7	51.3	29.0	24.4	8.3
AP2184, Wk8	0.218		34.6	26.0	45.7	67.2	51.3	10.4
AP2184, Wk16	0.256	10	29.9	27.3	34.1	46.6	46.2	12.2
AP2184, Wk24	0.363	10	27.1	26.7	38.3	34.5	34.3	8.6
AP2184, Wk32	0.040		20.0	23.1	24.0	28.9	33.9	7.7
AP2184, Wk40	0.264	7	22.3	27.9	27.4	31.0	46.5	8.3
AP2184, Wk45	-0.002	12	22.5	27.9	38.3	46.1	64.2	9.8
AP2184, Wk47		10	19.6	23.5	34.1	45.7	63.6	8.7
AP2185, Wk0	0.050	4	18.9	22.9	33.6	19.8	28.9	8.1
AP2185, Wk4	0.032		21.7	23.9	30.7	28.3	24.2	8.8
AP2185, Wk8	0.005		20.5	26.7	29.9	45.3	42.6	9.7
AP2185, Wk16	0.294	10	20.5	24.8	30.6	66.6	89.8	9.2
AP2185, Wk24	0.369	7	22.2	28.0	28.5	59.8	76.0	8.6
AP2185, Wk32	0.654		23.1	28.4	26.3	53.1	87.5	8.7
AP2185, Wk40	0.062	15	21.5	28.2	24.4	44.2	68.9	9.5
AP2185, Wk45	0.130	9	18.7	25.6	25.6	45.8	59.5	8.8
AP2185, Wk47		14	20.8	26.5	24.1	43.6	59.9	8.7
AP2188, Wk0	0.193	0	25.2	25.0	95.1	38.8	45.8	15.0
AP2188, Wk4	0.310		24.5	28.5	96.9	49.9	40.8	12.1
AP2188, Wk8	0.161		23.6	29.2	86.8	59.3	64.8	13.2
AP2188, Wk16	0.059	15	33.6	28.1	56.7	80.9	96.2	25.7
AP2188, Wk24	-0.001	4	35.1	29.7	56.9	72.5	98.8	23.5
AP2188, Wk32	0.083		31.3	32.5	31.4	81.7	88.8	17.0
AP2188, Wk40	0.070	4	29.0	25.1	31.6	87.0	76.9	15.4
AP2188, Wk45	0.053	4	31.1	26.8	28.8	84.5	70.9	13.2
AP2188, Wk47		5	34.5	27.9	35.4	85.0	74.2	14.4
AP2189, Wk0	0.491	2	24.0	25.9	38.5	22.5	18.7	9.5
AP2189, Wk4	0.393		39.3	26.7	73.6	23.5	30.4	9.5
AP2189, Wk8	0.136		56.3	28.2	58.8	26.7	35.0	9.6
AP2189, Wk16	0.199	7	65.9	29.8	47.3	65.0	71.1	11.4

AP2189, Wk24	0.405	6	44.1	29.6	36.6	61.1	78.2	24.7
AP2189, Wk32	0.218		36.8	26.8	29.6	72.2	90.7	36.9
AP2189, Wk40	0.263	2	39.2	28.5	32.9	71.3	83.6	32.0
AP2189, Wk45	0.342	3	34.3	30.1	37.9	64.7	76.2	26.9
AP2189, Wk47		4	36.3	30.8	34.6	63.2	76.2	30.0

ELISA responses (% positive control) to six synthetic lipid antigens and sample information for APHA timeline samples (10 animals at 9 times). Columns 2 and 3 are results for commercial Bovigam IGRA and ParaTB IDEXX assays. * O.D values from a Bovigam IGRA assay, derived from overnight stimulation of whole blood with PPDA minus a Nil (RPMI only) value; PPDB assays were also carried out. An animal is considered bTB positive if the O.D values of PPDB-PPDA is greater than 0.1 O.D on the Bovigam ELISA; within the limits of the assay, none of these animals were above that value. No IGRA assays were carried out at week 47. ** Values of above 55 are considered positive; those between 45 and 55 are intermediate and those below 45 are negatives.

Table S3: **ELISA responses (% positive control) to six synthetic lipid antigens for APHA and Moredun samples**

Serum ID	MOD171	JRRR121	RT237F2	SMP70	ZAM295	ST123
	ELISA absorbance, % Pooled Pos					
Reactor 1	30.9	23.5	33.1	71.5	64.7	32.5
Reactor 2	48.2	21.1	36.1	89.1	84.8	73.7
Reactor 3	25.4	23.3	28.5	53.5	41.1	22.4
Reactor 4	31.6	23.7	26.2	46.3	70.2	25.1
Reactor 5	36.1	23.6	28.8	50.7	36.4	10.7
Reactor 6	31.4	21.5	94.0	42.0	49.7	14.2
Reactor 7	29.4	26.9	36.8	27.5	28.0	13.9
Reactor 8	37.0	28.4	38.5	41.0	79.8	51.7
Reactor 9	37.9	26.5	39.2	76.7	54.1	22.9
Reactor 10	30.0	24.2	57.7	73.8	66.2	23.3
Reactor 11	31.3	22.0	34.6	35.5	66.0	47.6
Reactor 12	40.8	24.7	42.9	82.4	40.6	11.0
Reactor 13	204.1	22.2	61.3	46.7	41.5	8.7
Reactor 14	26.2	20.4	35.0	70.3	62.7	25.1
Reactor 15	21.4	19.7	23.9	19.9	59.4	15.9
Reactor 16	26.3	22.2	28.2	43.4	34.6	8.6
Reactor 17	25.3	22.1	27.9	37.9	27.7	8.6
Reactor 18	49.7	22.2	25.5	65.3	89.1	20.2
Reactor 19	46.1	24.1	57.3	54.8	64.9	43.0
Reactor 20	30.4	27.6	39.1	72.9	81.0	26.0
Gudair 1	39.0	29.4	45.2	45.0	26.5	13.0
Gudair 2	35.5	23.5	138.7	25.1	23.5	8.9
Gudair 3	44.4	25.8	59.3	37.9	25.7	9.2
Gudair 4	266.3	25.1	72.0	60.5	39.3	9.8
Gudair 5	32.7	22.3	36.4	28.7	21.4	8.9
Gudair 6	34.7	22.6	38.4	41.9	50.9	8.1
Gudair 7	43.7	23.7	46.6	58.9	26.5	8.5
Gudair 8	40.8	25.8	58.5	55.6	25.4	9.8
Gudair 9	149.0	30.6	44.6	73.5	62.6	16.7
Gudair 10	40.5	21.4	68.6	34.2	38.1	8.3
Gudair 11	64.9	24.0	129.5	80.5	63.5	15.0
Gudair 12	46.1	29.8	73.1	53.8	52.3	13.8
Gudair 13	106.6	25.9	65.6	52.0	58.4	15.5
Gudair 14	47.0	23.3	171.5	62.1	65.7	12.1
Gudair 15	49.1	25.0	52.2	62.0	88.0	69.9
Gudair 16	44.8	31.2	58.3	73.9	55.3	12.2
Gudair 17	103.7	25.6	57.4	46.5	34.0	9.9
Gudair 18	61.1	29.8	78.3	77.6	86.1	56.6
Gudair 19	51.8	26.3	39.4	56.3	52.8	11.0

Gudair 20	36.6	26.8	68.5	81.6	71.7	15.1
BCG Vac 1	25.0	24.4	43.5	16.6	15.0	8.8
BCG Vac 2	32.8	24.8	36.4	84.0	27.2	9.8
BCG Vac 3	31.1	26.8	58.5	58.3	23.6	9.4
BCG Vac 4	32.7	32.5	51.5	88.6	37.9	11.5
BCG Vac 5	21.1	24.9	30.5	14.3	12.0	9.4
BCG Vac 6	77.2	25.5	41.8	54.1	36.3	9.4
BCG Vac 7	23.3	23.0	128.4	48.1	32.9	10.3
BCG Vac 8	28.8	23.8	73.4	18.1	48.2	12.8
BCG Vac 9	23.8	23.1	65.0	91.2	18.2	8.5
BCG Vac 10	22.2	23.9	33.9	40.2	14.4	7.9
BCG Vac 11	21.3	21.5	41.3	14.6	20.1	7.4
BCG Vac 12	24.3	25.3	47.4	13.5	24.8	8.6
BCG Vac 13	20.2	21.3	105.3	19.3	18.3	8.7
BCG Vac 14	19.9	20.8	141.4	16.3	24.5	8.2
BCG Vac 15	31.3	22.9	63.0	100.1	54.4	9.0
BCG Vac 16	48.6	26.4	65.4	43.0	54.0	10.2
BCG Vac 17	29.4	26.6	49.0	40.7	69.8	16.1
BCG Vac 18	25.9	24.3	44.3	37.0	66.6	14.4
BCG Vac 19	35.7	23.0	69.4	29.5	33.6	7.6
BCG Vac 20	46.1	29.3	58.1	74.0	64.5	8.9
BCG Vac-Inf 1	126.3	23.1	46.5	51.5	63.8	11.1
BCG Vac-Inf 2	28.3	30.3	140.9	47.2	61.9	13.6
BCG Vac-Inf 3	24.6	21.8	98.4	31.0	82.8	50.8
BCG Vac-Inf 4	36.5	28.6	45.6	42.9	73.6	26.5
BCG Vac-Inf 5	31.7	27.6	52.2	69.2	59.2	10.4
BCG Vac-Inf 6	32.1	25.8	54.6	54.5	59.2	10.9
BCG Vac-Inf 7	39.1	25.3	52.4	93.2	34.5	8.5
BCG Vac-Inf 8	38.9	30.3	99.9	53.4	62.6	11.8
BCG Vac-Inf 9	28.5	23.5	46.8	94.3	89.4	72.0
BCG Vac-Inf 10	50.9	22.6	57.7	106.1	94.8	101.9
BCG Vac-Inf 11	21.1	22.4	41.8	54.6	47.4	8.1
BCG Vac-Inf 12	26.7	31.9	46.6	39.9	64.4	14.2
BCG Vac-Inf 13	42.7	28.7	46.4	79.7	31.0	9.7
BCG Vac-Inf 14	25.8	23.5	42.4	30.4	72.6	43.7
BCG Vac-Inf 15	31.7	24.3	44.3	43.2	41.5	8.4
BCG Vac-Inf 16	56.6	29.3	45.6	97.7	93.6	85.1
BCG Vac-Inf 17	149.2	26.2	56.1	58.2	45.4	10.2
BCG Vac-Inf 18	33.9	25.6	56.7	47.7	72.7	16.4
BCG Vac-Inf 19	27.6	23.6	37.9	75.3	44.7	10.8
BCG Vac-Inf 20	41.7	29.8	46.9	99.2	61.1	17.9
Control 1	31.8	26.1	41.9	87.8	66.8	38.4
Control 2	27.0	23.0	38.8	55.3	83.1	50.4
Control 3	41.7	27.2	90.1	75.0	63.7	27.3
Control 4	39.4	31.0	61.4	26.6	27.1	10.3
Control 5	41.3	26.3	63.9	66.3	53.5	13.2

Control 6		28.8	25.7	58.1	53.5	21.7	9.8
Control 7		27.4	32.0	46.1	54.6	46.4	11.1
Control 8		45.2	34.8	80.9	71.2	79.0	22.5
Control 9		39.1	27.2	115.0	85.6	45.4	12.2
Control 10		32.4	23.9	34.7	81.6	40.6	9.5
Control 11		33.3	24.4	126.2	29.8	53.6	9.9
Control 12		38.7	28.6	39.0	47.8	80.9	37.9
Control 13		37.7	29.2	56.5	45.3	60.0	10.2
Control 14		26.8	23.3	71.6	69.5	19.5	7.4
Control 15		29.4	24.7	45.7	19.3	24.9	7.9
Control 16		37.0	34.7	60.2	59.2	32.8	8.9
Control 17		33.1	33.5	98.6	35.0	28.1	9.7
Control 18		28.6	25.1	41.0	17.6	31.8	7.8
Control 19		29.6	23.0	37.9	16.6	23.0	7.6
Control 20		31.9	25.0	37.9	68.3	49.0	10.3
MRI153	Moredun	22.2	17.8	49.1	34.0	63.7	21.3
MRI156		22.2	18.2	82.6	50.2	35.7	10.7
MRI397		22.8	17.7	49.7	48.0	79.6	44.9
MRI398		24.4	17.8	43.9	57.6	73.4	38.3
MRI402		17.2	17.5	38.4	28.1	63.1	17.6
MRI403		29.2	27.4	54.7	63.5	62.7	11.1
MRI404		24.7	15.7	48.9	36.8	70.4	23.5
MRI405		19.7	16.6	39.7	35.8	45.2	13.2
MRI406		28.9	20.0	50.4	35.5	84.0	6.6

ELISA responses (% positive control) to six synthetic lipid antigens for APHA and Moredun samples

Figure S1 Structures of synthetic antigens used in this work

MOD171

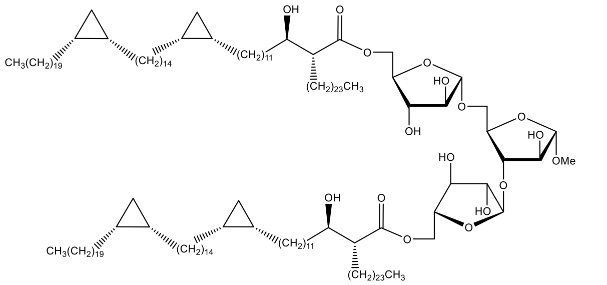

SMP70

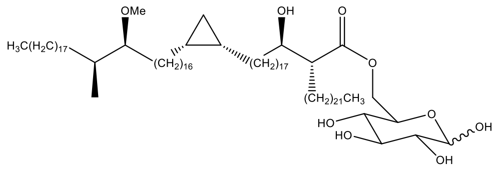

ZAM295

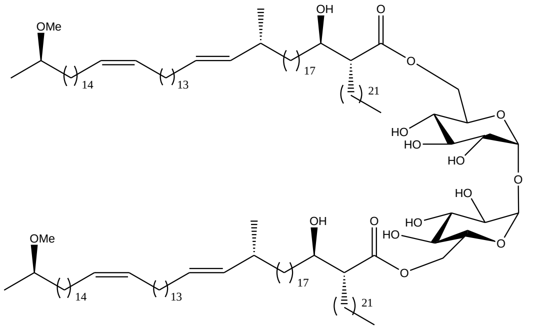

RT237-F2

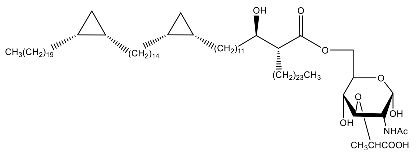

ST123

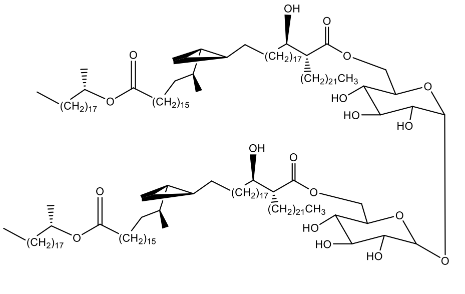

JRRR121

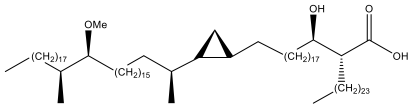

Table S4: Median ELISA responses for each set of samples with each of six antigens as % positive control for that antigen.

		MOD 171	JRRR 121	RT237 F2	SMP 70	ZAM 295	ST 123		ST, ZAM	ST, JR	ST, JR, ZAM	JR,SMP, ZAM	All Six Antigen
		Absorbance % Pooled Pos							Combined Antigen Positive Probability (0 to 1)				
PEI++	Median +	27.3	38.8	56.5	88.4	96.3	79.0		0.9	0.9	0.9	0.9	0.8
F5 plus F6	Median -	24.7	17.4	48.8	54.2	50.7	23.7		0.1	0.1	0.1	0.1	0.1
	ROC AUC	0.55	0.88	0.64	0.82	0.95	0.9		0.91	0.91	0.94	0.92	0.93
	ROC threshold	33.8	24.8	49.2	74.1	88.9	27.0		61.4	64.5	42.5	53.3	44.6
	Sensitivity	44.8	81.3	75.0	75.0	87.5	100.0		87.5	75.0	93.8	87.5	93.8
	Specificity %	78.8	85.0	51.3	80.0	92.5	61.3		90.0	97.5	87.5	92.5	86.3
	**Cut-off for >93% specificity**	57	40	76	93	91	68		0.8	0.63	0.73	0.63	0.78
	**Sensitivity %**	25	50	6.25	37.5	75	62.5		56.25	75	68.75	68.75	68.75
	**Specificity %**	95.0	96.3	93.8	95.0	93.8	93.8		93.8	97.5	97.5	97.5	100.0
	**Accuracy**	60.0	73.1	50.0	66.3	84.4	78.1		75.0	86.3	83.1	83.1	84.4
F5	Median -	24.8	19.4	55.2	43.5	50.7	21.4		0.0	0.1	0.1	0.1	0.1
Negatives	Cut-off	57	40	76	93	91	68		0.8	0.63	0.73	0.63	0.78
	Specificity %	97.5	92.5	92.5	95	95	97.5		97.5	100	97.5	100	100
F6	Median -	24.6	15.4	45.0	64.5	51.2	27.9		0.1	0.2	0.1	0.1	0.1
Negatives	Cut-off	57	40	76	93	91	68		0.8	0.63	0.73	0.63	0.78
	Specificity %	92.5	100	95	95	92.5	90		90	95	97.5	95	100

Median ELISA responses for each set of samples with each of six antigens as % positive control for that antigen. Optimal cut-offs are based on 16 PEI culture and IDEXX positive samples (PEI++) compared to all 80 PEI F5 and F6 negatives (again as % positive control), and sensitivity and specificity of each group based on those cut-offs. ROC analysis of these data is presented graphically in Fig S2. In some cases, such as for MOD171, the optimal cut-off by ROC analysis led to a very low specificity; cut-offs were then identified to give maximum accuracy (average of sensitivity and specificity) at >93 % specificity. The final five columns show the median calculated negative or positive status (on 0 to 1 scale) of PEI++ compared to all 80 F5 and F6 negative samples, combining results with several specified antigens using the R statistics package. These calculated values for each serum sample have then been analysed by ROC (shown graphically in Fig S2) and optimal cut-offs determined to provide the highest accuracy (average of sensitivity and specificity). Again, where the ROC analysis gave a specificity of below 93%, the cut-off was adjusted to meet that value. The data for F5 and F6 samples are also presented separately, using the same cut-offs.

Figure S2: ROC analysis of ELISA responses for PEI++ samples compared to 80 F5 and F6 negatives

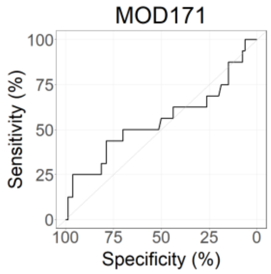


```
Optimal Cutoff
  threshold specificity sensitivity
1    33.805       78.75       43.75

Area under the curve: 55.16%
Confidence Interval
95% CI: 36.86%-73.45% (DeLong)
```

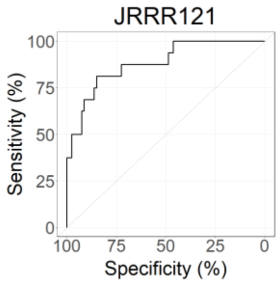


```
Optimal Cutoff
  threshold specificity sensitivity
1     24.83          85       81.25

Area under the curve: 88.12%
Confidence Interval
95% CI: 78.87%-97.38% (DeLong)
```

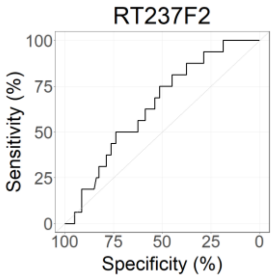


```
Optimal Cutoff
  threshold specificity sensitivity
1    48.035       45.00       81.25
2    49.165       51.25       75.00

Area under the curve: 64.34%
Confidence Interval
95% CI: 50.85%-77.82% (DeLong)
```

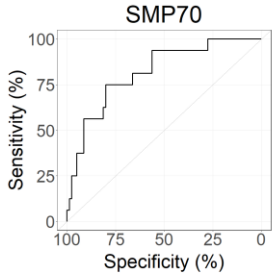


```
Optimal Cutoff
  threshold specificity sensitivity
1    74.105          80          75

Area under the curve: 81.56%
Confidence Interval
95% CI: 70.44%-92.69% (DeLong)
```

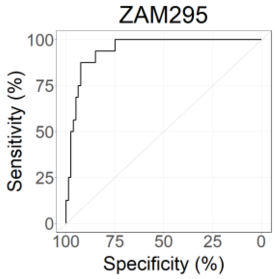


```
Optimal Cutoff
  threshold specificity sensitivity
1    88.925        92.5        87.5

Area under the curve: 94.53%
Confidence Interval
95% CI: 89.96%-99.1% (DeLong)
```

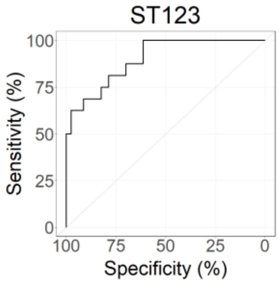


```
Optimal Cutoff
  threshold specificity sensitivity
1     26.96       61.25         100

Area under the curve: 90%
Confidence Interval
95% CI: 82.23%-97.77% (DeLong)
```

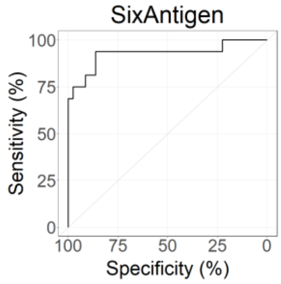


```
Optimal Cutoff
  threshold specificity sensitivity
1 0.4456839       86.25       93.75

Area under the curve: 92.73%
Confidence Interval
95% CI: 83.11%-100% (DeLong)
```

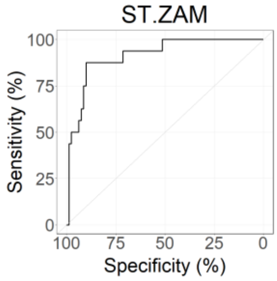


```
Optimal Cutoff
  threshold specificity sensitivity
1 0.6136764          90        87.5

Area under the curve: 91.25%
Confidence Interval
95% CI: 84.03%-98.47% (DeLong)
```

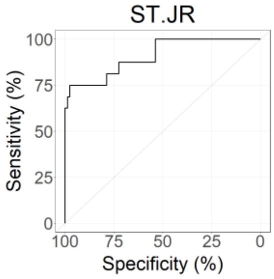


```
Optimal Cutoff
  threshold specificity sensitivity
1 0.6446451        97.5          75

Area under the curve: 90.94%
Confidence Interval
95% CI: 82.42%-99.45% (DeLong)
```

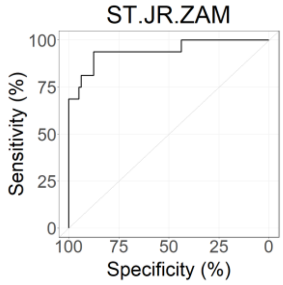


```
Optimal Cutoff
  threshold specificity sensitivity
1 0.4253513        87.5       93.75

Area under the curve: 94.22%
Confidence Interval
95% CI: 87.07%-100% (DeLong)
```

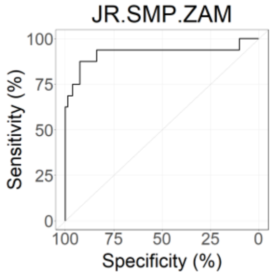


```
Optimal Cutoff
  threshold specificity sensitivity
1 0.5332295        92.5        87.5

Area under the curve: 92.11%
Confidence Interval
95% CI: 81.05%-100% (DeLong)
```

Figure S3: Box plots showing distribution of ELISA responses for each group of samples PEI++, F5 and F6 when combined in R as in Tables 1 and S1.

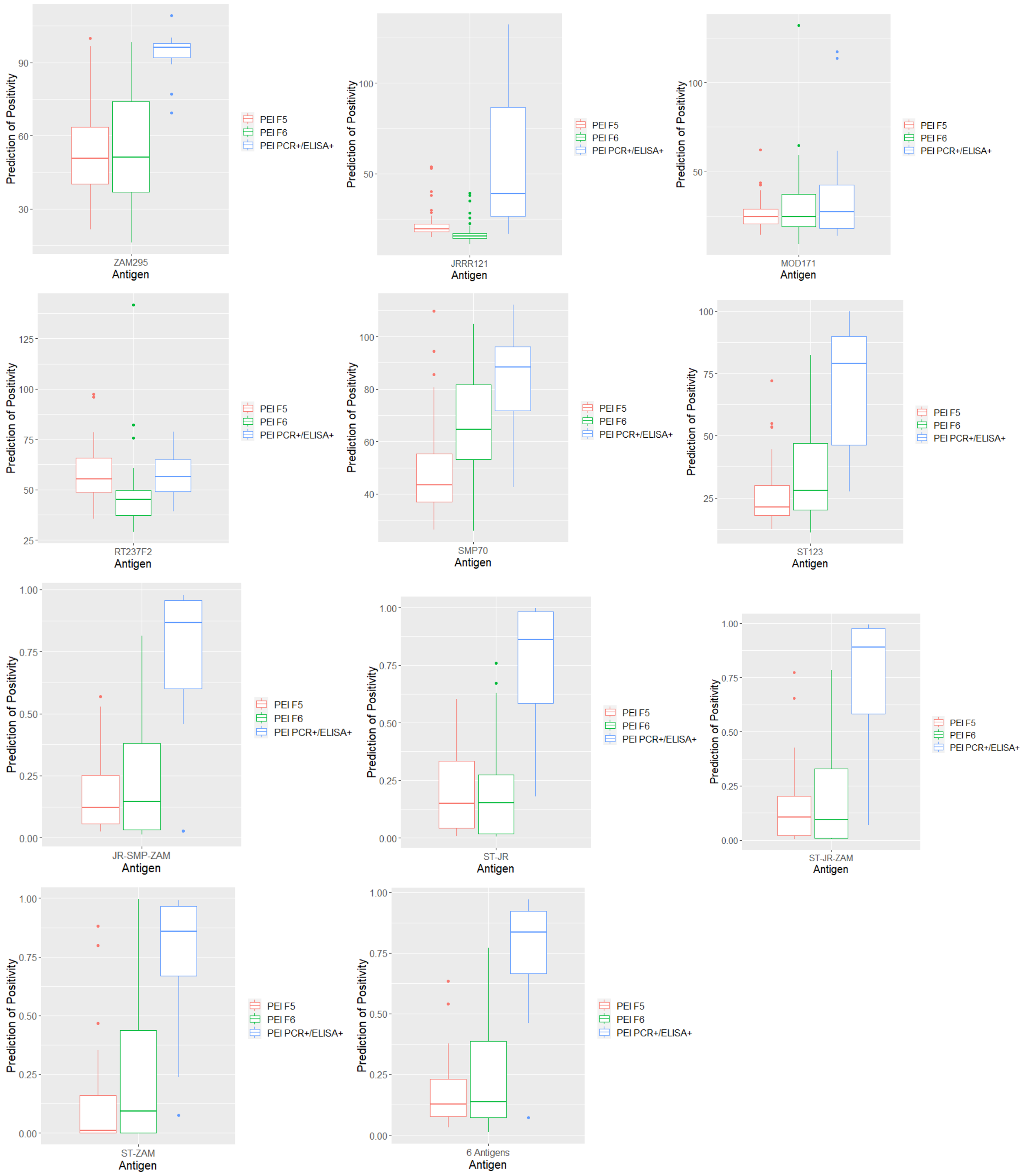

Table S5: Median ELISA responses (as % positive control) and sensitivity/specificity for Moredun 'no history' samples and for each cohort of APHA serum samples, using the same cut-offs as determined in Table 1.

			MOD 171	JRRR 121	RT237 F2	SMP 70	ZAM 295	ST 123	ST, ZAM	ST, JR	ST, JR, ZAM	JR, SMP, ZAM	All Six Antigen
			Absorbance % Pooled Pos						Probability				
All negatives (PEI F5/F6, controls, BCG/Gudair, timeline, moredun		Median -	27.7	24.7	45.7	50.3	52.9	14.0	0.0	0.2	0.1	0.2	0.1
n = 239		Cut-off	57	40	76	93	91	68	0.8	0.63	0.73	0.63	0.78
		Sensitivity %	25.0	50.0	6.3	37.5	75.0	62.5	56.3	75.0	68.8	68.8	68.8
		Specificity %	94.6	98.3	90.0	96.7	94.6	96.2	95.8	94.6	96.2	92.5	99.2
Reactors		Median -	31.4	23.4	35.5	52.1	61.1	22.6	0.1	0.2	0.1	0.2	0.2
n = 20		Specificity %	95.0	100.0	95.0	100.0	100.0	95.0	100.0	90.0	100.0	100.0	100.0
Gudair vac		Median -	45.4	25.7	58.9	55.9	51.6	11.5	0.0	0.2	0.1	0.1	0.1
n = 20		Specificity %	70.0	100.0	80.0	100.0	100.0	95.0	100.0	90.0	95.0	90.0	100.0
BCG Vac		Median -	27.4	24.4	54.8	40.5	30.0	9.2	0.0	0.1	0.1	0.1	0.1
n = 20		Specificity %	95.0	100.0	85.0	95.0	100.0	100.0	100.0	100.0	100.0	100.0	100.0
BCG Vac-Inf		Median -	33.0	25.7	46.9	54.6	62.2	12.7	0.1	0.2	0.1	0.3	0.2
n = 20		Specificity %	90.0	100.0	85.0	75.0	90.0	85.0	90.0	90.0	85.0	85.0	90.0
APHA Control		Median -	32.7	26.2	57.3	54.9	45.9	10.3	0.0	0.2	0.1	0.2	0.1
n = 20		Specificity %	100.0	100.0	75.0	100.0	100.0	100.0	100.0	100.0	100.0	95.0	100.0
Moredun serum		Median -	22.8	17.8	49.1	36.8	63.8	17.6	0.1	0.1	0.1	0.2	0.2
n = 9		Specificity %	100.0	100.0	88.9	100.0	100.0	100.0	100.0	100.0	100.0	100.0	100.0
Timeline		Median -	31.8	25.0	53.7	51.0	51.2	10.6	0.0	0.2	0.1	0.2	0.1
n = 90		Specificity %	97.8	98.9	93.3	96.7	91.1	96.7	94.4	90.0	93.3	85.6	97.8
PEI+/-		Median+ -	23.5	21.2	56.5	64.0	66.6	29.8	0.2	0.3	0.3	0.3	0.3
n = 64		Specificity %	95.3	87.5	75.0	93.8	79.7	87.5	82.8	76.6	87.5	81.3	95.3
		Positives	3	8	16	4	13	8	11	15	8	12	3

Median ELISA responses (as % positive control) and sensitivity/specificity for Moredun 'no history' samples and for each cohort of APHA serum samples, using the same cut-offs as determined in Table 1. PEI++ data show the corresponding sensitivity with the 16 PEI samples both positive to culture and giving IDEXX responses above the recommended cut-off for MAP infection. Specificity values for single antigens are determined from Table S4 using the cut-offs from Table 1. Specificity values for combinations of antigens using R for each cohort are calculated using the antigen

combinations determined in Table 1; the cut-off for R calculations are as in Table 1 and are on a scale 0 (negative) to 1 (positive), applied to the data in Tables S6 and S7

Figure S4: Box plots showing distribution of ELISA responses for each group of samples PEI++, F5 and F6 compared to Moredun and APHA cohorts

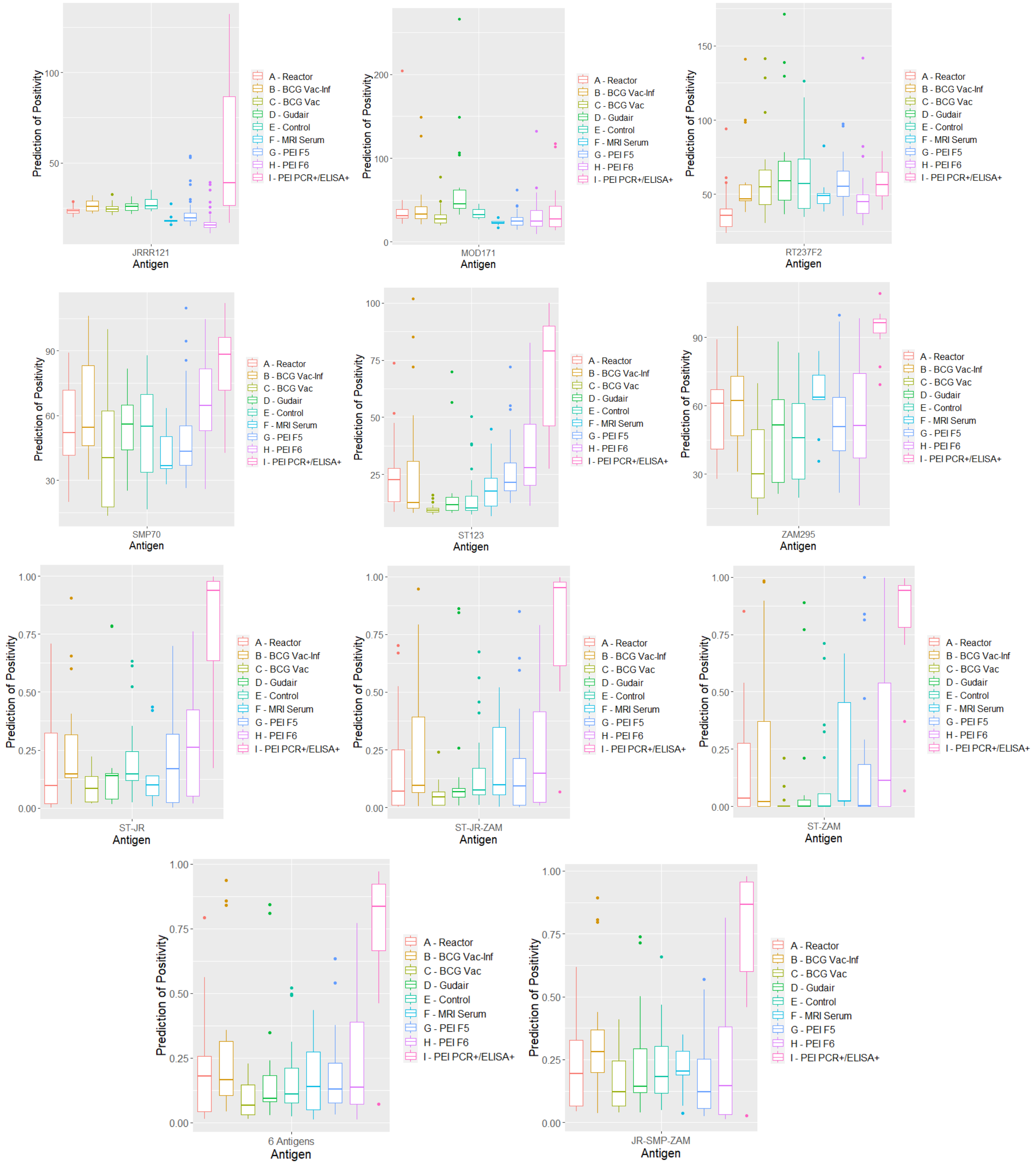

Table S6. Analysis of all 80 PEI PCR samples giving non-zero cycle counts (Table S1) with cut-offs optimised for maximum accuracy compared to all 80 F5 and F6 negatives.

		Cut-off	56	38	94	90	94	65	0.75	0.68	0.71	0.69	0.7	0.7
			MOD 171	JRRR 121	RT237 F2	SMP 70	ZAM 295	ST 123	ST, ZAM	ST, JR	SMP. JR, ZAM	ST, JR, ZAM	Six Antigen	ST, JR, SMP
			Absorbance % Pooled Pos						Probability of positive (0 to 1)					
		Sensitivity %	10.0	20.0	8.8	12.5	22.5	26.3	22.5	37.5	38.8	38.8	36.3	28.75
F5F6		Specificity %	92.5	92.5	96.3	91.3	95.0	91.3	95.0	97.5	95.0	95.0	95.0	96.25
F5		Specificity %	95	97.5	95	95	92.5	87.5	92.5	97.5	92.5	92.5	95	100
F6		Specificity %	90	87.5	97.5	87.5	97.5	95	97.5	97.5	95	97.5	95	92.5
All, minus APHA Reactors and BCG Vac-Inf		Specificity %	93.3	97.1	92.9	94.6	95.8	95.0	96.2	91.2	87.0	85.8	95.4	92.1
Reactor		Specificity %	95.0	100.0	100.0	100.0	100.0	95.0	100.0	85.0	90.0	90.0	100.0	90.0
Gudair		Specificity %	70.0	100.0	85.0	100.0	100.0	95.0	100.0	90.0	90.0	90.0	95.0	95.0
BCG Vac		Specificity %	95.0	100.0	85.0	90.0	100.0	100.0	100.0	100.0	100.0	100.0	100.0	100.0
BCG Vac-Inf		Specificity %	85.0	100.0	85.0	75.0	95.0	85.0	90.0	80.0	85.0	80.0	90.0	85.0
Control		Specificity %	100.0	100.0	85.0	100.0	100.0	100.0	100.0	85.0	95.0	85.0	90.0	85.0
TL		Specificity %	96.7	98.9	94.4	95.6	93.3	95.6	94.4	84.4	74.4	72.2	95.6	86.7
Moredun serum		Specificity %	100.0	100.0	100.0	100.0	100.0	100.0	100.0	100.0	100.0	100.0	100.0	100.0

Analysis of all 80 PEI PCR samples giving non-zero cycle counts (Table S1) with cut-offs optimised for maximum accuracy compared to all 80 F5 and F6 negatives. Cut-offs adjusted to give a minimum specificity with each antigen of 91 %, and with each antigen combination in R of 92.5 %. Data for F5 and F6 are then presented separately. Data for other cohorts of cattle analysed to the same cut-offs. Results with each other sub-set then analysed to same cut-offs and same antigen combinations in the R statistics package.
